# Clinical features, hospitalisation and deaths associated with monkeypox: a systematic review and meta-analysis

**DOI:** 10.1186/s12941-022-00527-1

**Published:** 2022-08-10

**Authors:** Vicente A. Benites-Zapata, Juan R. Ulloque-Badaracco, Esteban A. Alarcon-Braga, Enrique A. Hernandez-Bustamante, Melany D. Mosquera-Rojas, D. Katterine Bonilla-Aldana, Alfonso J. Rodriguez-Morales

**Affiliations:** 1 Escuela de Medicina, Universidad Peruana de Ciencias Aplicadas, Lima, Perú; 2 Sociedad Científica de Estudiantes de Medicina de la Universidad Peruana de Ciencias Aplicadas, Lima, Perú; 3 Sociedad Científica de Estudiantes de Medicina de la Universidad Nacional de Trujillo, Trujillo, Perú; 4 Grupo Peruano de Investigación Epidemiológica, Unidad Para la Generación y Síntesis de Evidencias en Salud, Universidad San Ignacio de Loyola, Lima, Perú; 5 Grupo de Investigación Biomedicina, Faculty of Medicine, Fundación Universitaria Autónoma de las Américas, Pereira, Risaralda Colombia; 6 Latin American Network of MOnkeypox VIrus Research (LAMOVI), Pereira, Risaralda Colombia; 7 Master of Clinical Epidemiology and Biostatistics, Universidad Científica del Sur, Lima, Perú; 8 Faculty of Medicine, Institución Universitaria Visión de las Américas, Pereira, Risaralda, Colombia

**Keywords:** Monkeypox, Orthopoxvirus, Poxviridae, Zoonotic, Clinical features, Laboratory, Outcomes, Epidemic

## Abstract

**Introduction:**

A multicountry monkeypox disease (MPX) outbreak began in May 2022 in Europe, leading to the assessment as a potential Public Health Emergency of International Concern (PHEIC) on June 23, 2022. Some observational studies have partially characterised clinical features, hospitalisations, and deaths. However, no systematic reviews of this MPX outbreak have been published.

**Methods:**

We performed a systematic review with meta-analysis, using five databases to assess clinical features, hospitalisations, complications and deaths of MPX confirmed or probable cases. Observational studies, case reports and case series, were included. We performed a random-effects model meta-analysis to calculate the pooled prevalence and 95% confidence interval (95% CI). In addition, we carried out a subgroup analysis according to the continents and a sensitivity analysis excluding studies classified as having a high risk of bias.

**Results:**

A total of 19 articles were included, using only 12 articles in the quantitative synthesis (meta-analysis). For 1958 patients, rash (93%, 95% CI 80–100%), fever (72%, 95% CI 30–99%), pruritus (65%, 95% CI 47–81%), and lymphadenopathy (62%, 47–76%), were the most prevalent manifestations. Among the patients, 35% (95% CI 14–59%) were hospitalised. Some 4% (95% CI 1–9%) of hospitalised patients had fatal outcomes (case fatality rate, CFR).

**Conclusion:**

MPX is spreading rapidly, with a third of hospitalised patients, but less than 5% with fatal outcomes. As this zoonotic virus spreads globally, countries must urgently prepare human resources, infrastructure and facilities to treat patients according to the emerging guidelines and the most reliable clinical information.

**Supplementary Information:**

The online version contains supplementary material available at 10.1186/s12941-022-00527-1.

## Introduction

### Rationale

Monkeypox virus (MPXV), an Orthopoxvirus, is a genus that includes the smallpox virus [1, 2]. This emerging zoonotic agent was discovered in 1958 in Denmark among laboratory monkeys but had been causing human disease since 1970 in endemic countries in Africa [3–6]. This pathogen causes a two-clinical stage illness, including a prodrome during its invasive period and a cutaneous phase defined as the skin eruption [7]. Although described over decades, the current ongoing outbreak outside Africa seems to present atypical clinical manifestations compared to cases reported before 2022 in endemic countries [8–10]. The MPXV, taxonomically, is currently part of the genus Orthopoxvirus, which belongs to the subfamily Chordopoxvirinae in the family Poxviridae. That family is part of the order Chitovirales included in the class Pokkesviricetes. This class belongs to the phylum Nucleocytoviricota, in the kingdom Bamfordvirae, in the realm Varidnaviria [11–14].

Members of the genus Orthopoxvirus cause disease in humans and animals, as well as other members of the family Poxviridae affecting birds, goats, cervids, crocodiles, rabbits, and insects [6]. In the case of smallpox, this epidemic disease was eradicated by the 1980s after a successful global vaccination campaign. Since there, no vaccination against smallpox has continued [15]. As expected, several similarities and differences in the epidemiology, clinical features, and management of smallpox and monkeypox have been identified. These are enveloped double‐stranded DNA viruses with a genome ranging from 130 to 300 kbp (around 190 kbp for monkeypox) [16]. Therefore, a complete clinical characterisation of MPX disease, as well as cutaneous lesions, hospitalisation and outcome, is required.

Although only two months have elapsed since the global spreading of MPX in 2022 [17], some studies and case reports have been already published in major international scientific and medical journals from European and other countries with travel- and non-travel-related cases [8, 9, 18, 19]. Many of these reports have started to answer clinical questions, including evolution and outcomes, potential risk factors, and clinical, especially dermatological findings [20]; however, a systematic review consolidates what has been learned from each study or reported case is to date missing. Although systematic reviews and meta-analyses usually include randomised clinical trials (RCTs) and aim to provide a more precise estimate of the effect of a treatment or risk factor for disease, they also have been extensively used, especially during the last decades, to synthesised observational studies [21–23]. However, RCTs are not feasible or available in many situations, and only data from observational studies are accessible [23]. That is the case for the clinical, hospitalisation, and outcome features of MPX.

### Objectives

- To summarise the clinical features of MPX reported in currently available observational studies.
- To assess the clinical spectrum of the cutaneous manifestations and their frequency.
- To examine the outcome of MPX cases, including the proportion of patients requiring hospitalisation and those with fatal outcomes.

## Methods

### Protocol and registration

This protocol follows the recommendations established by the Preferred Reporting Items for Systematic Reviews and Meta-Analyses (PRISMA) statement [24], and it has been reported in the International Prospective Register of Systematic Reviews (PROSPERO) database (CRD42022336855).

### Databases and search strategy

A comprehensive search about the prevalence of clinical manifestations, characteristics of the lesions and complications of patients diagnosed with monkeypox was conducted on June 7, 2022, through the following databases: PubMed, Scopus, Embase, Ovid-Medline and Web of Science. No restrictions regarding language or publication date were applied. The search strategy was built using the Peer Review of Electronic Search Strategies (PRESS) Checklist, and we carried out a hand search of the reference lists of the included studies and preprints databases (The Lancet Preprints, medRxiv and ResearchSquare). The complete search strategy is available in Additional file 1: Table S1.

### Study selection and data extraction

This systematic review had a comprehensive scope, including the subsequent observational studies: cross-sectional, cohort, case–control, case reports and case series. In addition, studies assessing the prevalence of the various clinical manifestations, characteristics of the lesions and complications of patients with a probable or confirmed monkeypox diagnosis, regardless of age, were included. The definitions of possible and confirmed cases for each study are described in Additional file 1: Table S2. Case series with more than 10 cases were included in the qualitative and quantitative synthesis; however, studies with less than ten were included only in the qualitative synthesis. Scoping reviews, narrative reviews, systematic reviews and conference abstracts were excluded.

All the articles resulting from the electronic search were exported to the data management software “Rayyan QCRI”, and duplicate records were removed. Titles and abstracts were independently screened by four reviewers ( JRU-B, EAA-B, MDM-R and EAH-B). After identifying the potential references to be included, the reviewers independently assessed the full text of each article. Conflicts or discrepancies in decisions were resolved through debate among the total of the authors, then a consensus was reached. The data from the included articles were extracted through a data extraction sheet built in Microsoft Excel. The following information was extracted: author, year of publication, and the number of probable or confirmed cases affected by the clinical characteristics or complications. We used the Web-based tool “WebPlotDigitizer” to extract data from graphs in case it was not available in numerical format [25].

### Risk of bias assessment

We used the original Newcastle–Ottawa Scale (NOS) to assess the risk of bias in case–control and cohort studies, and the NOS was adapted for cross-sectional studies (NOS-CS). In both scales, a score of 7 or more stars was considered a low risk of bias, while a score of 6 or fewer stars was considered a high risk of bias. The quality assessment of case reports and case series were assessed with the Joanna Briggs Institute’s Checklist for Case Reports and Checklist for Case Series, respectively. As with the NOS and the NOS-C, the cut-off points for both checklists were seven stars. We assigned a star to every item answered as “Yes”; otherwise, it did not receive a star. In case multiple items were “Not applicable” for an article, the quality of the study was finally decided by consensus on a case-by-case basis. This examination was done independently by two reviewers (JRU-B and EAA-B).

### Assessment of publication bias

The publication bias assessment in proportional meta-analysis is an evaluation that is not recommended in the current literature. That is because conventional funnel plots and Egger’s test are inaccurate for these analyses. The reason behind this is that funnel plots were created assuming that studies with positive results were published more frequently when compared to studies with negative results; however, in a meta-analysis of proportions, there is no consensus on what a positive result is. Moreover, there is no evidence that proportions adjust correctly to funnel plots or Egger’s tests [26, 27].

### Statistical approach

The information collected from the included articles was combined using STATA 16.0. We conducted a pooled analysis of the various clinical manifestations, characteristics of the lesions and complications of patients with probable/confirmed monkeypox. A random-effects model (Dersimonian and Laird) was used for the quantitative analysis. The 95% Confidence Intervals for the proportions reported in each study were calculated using the Clopper-Pearson Method. The Freeman-Tukey Double Arcsine Transformation was used as the variance stabiliser. The Cochran’s Q test and the I^2^ statistic were used to assess the between-study-heterogeneity; values equal to or greater than 60% were classified as high heterogeneity for the I^2^ statistic, and a P-value < 0.05 was a sign of heterogeneity in the Cochran's Q test. In addition, we carried out a subgroup analysis according to the continent where the studies were conducted (when there were at least two studies to meta-analyse) and a sensitivity analysis excluding studies classified as having a high risk of bias.

## Results

### Study selection and characteristics

**Fig. 1:**
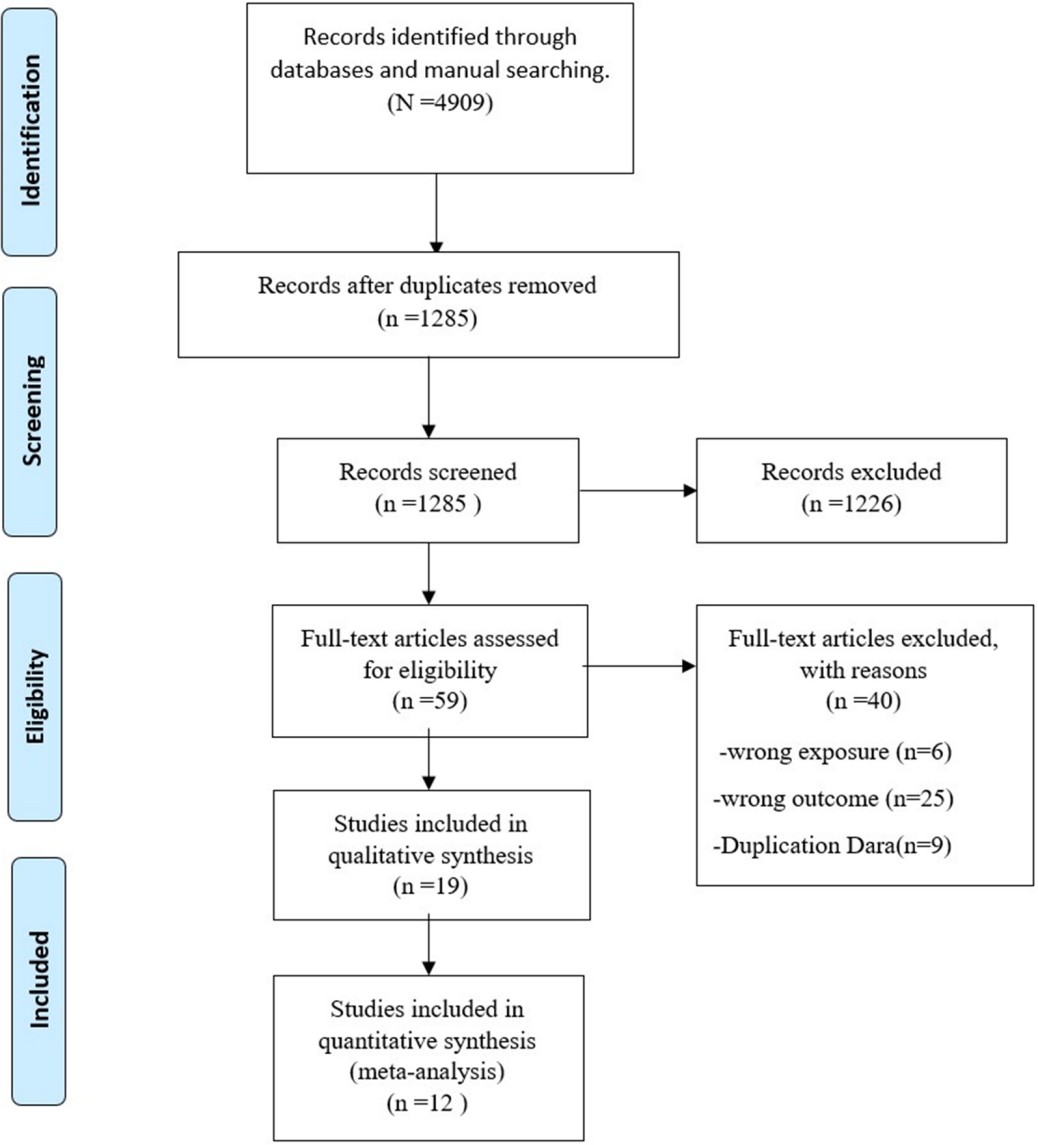
PRISMA Flow Diagram

The systematic search retrieved 4909 references across all databases, and duplicates were removed. Titles and abstracts screened the remaining 1285 references, and 59 articles remained for selection by full-text. After screening, 12 were included for quantitative synthesis (meta-analyses), and 19 studies were included in the qualitative synthesis [28–46]. The main characteristics of all included studies are summarised in Tables 1 and 2, and the study selection process is briefly described in the PRISMA flow diagram (Fig. 1).

**Table 1:** Characteristics and main clinical manifestations of the included studies

Author	Country	Year	Participants(male/female)	Median/mean /range age (IQR/SD)	Classification of the cases presented	Smallpox vaccination history (includes visible smallpox scar)
Formenty et al.	Sudan	2010	19 (9/10)	8 months–32 years	Confirmed cases: 10 Probable cases: 9	NR
Huhn et al.	USA	2005	34 (18/16)	26 (6–47)	All patients are confirmed cases	7/34
Yinka-Ogunleye et al.	Nigeria	2019	122 (84/38)	29 (2 days old-50 y),	Confirmed cases: 118 Probable cases: 4	NR
Whitehouse et al.	Democratic Republic of Congo	2021	1057 (568/469)	14 (6–23.9)	All patients are confirmed cases	97/1057
Ježek et al.	Democratic Republic of Congo	1988	338 (182/156)	3 months–69 years	All patients are confirmed cases	43/338
Kalthan et al.	Central African Republic	2016	12 (6/6)	31.6 (12.37)	Confirmed cases:4 Probable cases:8	NR
Pittman et al.	Democratic Republic of Congo	2022	216 (138/78)	14 (9.9)	All patients are confirmed cases	4/216
Breman et al.	Central and West Africa (5 countries)	1980	47 (21/26)	8 years (7 months-35 years)	Confirmed cases:35, Probable cases:12	4/47
Perez-Duque et al.	Portugal	2022	27 (27/0)	33 (22–59)	All patients are confirmed cases	1/27
Learned et al.	Democratic Republic of Congo	2003	11 (8/3)	9.6 (7.3)	Confirmed cases:3, Probable cases:8	0/11
Mande et al.	Democratic Republic of Congo	2022	21 (14/7)	16 (4–30)	All patients are confirmed cases	NR
Girometti et al.	United Kingdom	2022	54 (54/0)	41 (34- 45)	All patients are confirmed cases	NR
Vaughan et.al	United Kingdom	2018	2 (2/0)	NR	All patients are confirmed cases	NR
Antinori et al.	Italy	2022	4 (4/0)	NR	All patients are confirmed cases	1/4
Hammerschlag et al.	Australia	2022	1 (1/0)	NR	Confirmed Case	NR
De Nicolas-Ruanes et al.	Spain	2022	1 (1/0)	30	Confirmed Case	NR
Reynolds et al.	Sierra Leone	2019	1 (1/0)	35	Confirmed Case	NR
Erez et al.	Israel	2019	1 (1/0)	NR	Confirmed Case	NR
Adler et al.	United Kingdom	2022	7 (4/3)	NR	All patients are confirmed cases	NR

Clinical manifestations (affected patients/total patients)														
Lymphadenopathy	Photophobia	Myalgias	Fever	Arthralgia	Pruritus	Conjunctivitis	Rash	Diarrhoea	Cough	Headache	Fatigue	Sore throat	Difficulty breathing	Nausea or vomiting
15/19	NR	15/19	16/19	15/19	NR	11/19	19/19	2/9	10/19	11/19	12/19	NR	12/19	2/19
19/34	NR	19/34	28/34	2/34	NR	3/34	32/34	2/34	16/34	22/34	NR	21/34	6/34	11/34
45/65	NR	42/67	81/92	NR	57/78	NR	122/122	NR	NR	61/77	NR	45/77	NR	NR
876/1034	332/999	754/1003	1023/1057	NR	542/1012	210/1016	1057/1057	NR	561/1024	793/1011	888/1029	246/325	NR	250/1010
163/295	NR	NR	NR	NR	NR	NR	295/295	NR	100/280	NR	NR	NR	NR	NR
6/12	NR	NR	11/12	NR	NR	NR	12/12	NR	NR	2/12	NR	NR	NR	NR
213/216	5/216	15/216	1/216	21/216	NR	20/216	215/216	10/216	104/216	51/216	NR	169/216	15/216	23/216
18/45	NR	NR	NR	NR	NR	NR	NR	NR	NR	NR	NR	NR	NR	NR
14/27	NR	5/27	13/27	NR	NR	NR	14/27	NR	NR	7/27	NR	NR	NR	NR
2/11	7/8	NR	NR	NR	6/8	NR	8/11	NR	NR	NR	1/11	NR	NR	NR
10/21	NR	NR	20/21	NR	NR	NR	21/21	NR	NR	NR	NR	NR	NR	NR
30/54	NR	16/54	31/54	NR	NR	NR	6/54	NR	NR	N	36/54	6/54	NR	NR
2/2	NR	NR	2/2	NR	1/2	NR	2/2	NR	NR	NR	NR	NR	NR	NR
2/4	NR	1/4	2/4	NR	2/4	NR	4/4	NR	NR	NR	NR	NR	NR	NR
NR	NR	NR	1/1	NR	NR	NR	1/1	NR	NR	NR	NR	NR	NR	NR
1/1	NR	1/1	1/1	1/1	NR	NR	1/1	NR	NR	NR	NR	NR	NR	NR
1/1	NR	1/1	1/1	NR	NR	NR	1/1	NR	NR	NR	NR	NR	NR	NR
1/1	NR		1/1	NR	NR	NR	1/1	NR	NR	NR	NR	NR	NR	NR
5/7	NR	NR	NR	NR	1/7	1/7	7/7	NR	NR	NR	NR	2/7	NR	NR

IQR, interquartile range; SD, standard deviation; NR, not reported

**Table 2:** Characteristics of the rash and complications associated with MPX

Author	Rash (affected patients/total patients)				Lesions on mucous membranes (affected patients/total patients)	Number of lesions (affected patients/total patients)	Complications (affected patients/total patients)					
	Monomorphic	Pleomorphic	Rash site	Body Distribution			Ocular lesions	Secondary bacterial skin infection	Hemorrhagic pustules	Ulcerated or necrotic lesions	Deaths	Hospitalised patients
Formenty et.al.	NR	NR	Entire Body: 8/11 Head and/or neck: 3/8 Arms and/or hands: 3/8 Legs and/or feets: 3/8 Chest and/or Abdomen:	NR	Oral cavity: 6/8	< 100: 3/6 > 100:3/6	NR	NR	NR	NR	0/19	7/19
Huhn et.al.	21/31	9/31	Entire Body: 7/31 Head or neck: 20/32 Arms or hands: 26/32 Pelvic area and groin: 3/32 Legs or feet: 21/32 Chest or Abdomen:18/32	Centrifugal: 15/31 Centripetal:1/31	Oral cavity: 7/34	< 100: 24/30 > 100: 6/30	NR	19/40	2/34	8/34	0/34	9/34
Yinka-Ogunleye et al.	25/40	15/40	Head and/or neck: 68/71 Arms and/or hands: 55/70 pelvic area and groin: 44/65 Legs and/or feets: 63/69 Chest and/or Abdomen:56/70 Palms: 48/70 Sole of the foot: 42/66	NR	Genitals: 25/40	< 100: 16/40 > 100: 24/40	NR	19/40	NR	NR	7/122	NR
Whitehouse et al.	286/319	NR	Head and/or neck:1036/1057 Arms and/or hands: 1026/1057 Pelvic area and groin: 300/1057 Legs and/or feets: 786/1057 Chest and/or Abdomen:1028/1057 Palms: 1009/1057 v: 885/1057	Centrifugal: 989/1025 Centripetal: 33/1025	Oral cavity 570/1018 Genitals: 300/1057	< 100: 510/1043 > 100: 533/1043	NR	NR	NR	NR	NR	NR
Ježek et al.	233/295	62/295	Head and/or neck: 256/295 Palms: 206/295 Sole of the foot: 196/295	Centrifugal: 245/295 Centripetal: 13/295	Oral cavity: 207/295 Genitals: 88/295 Conjunctiva: 57/295	< 100: 77 /295 > 100: 218/295	11/295	48/295	0/295	NR	33/338	NR
Kalthan et.al	NR	NR	NR	NR	NR	NR	NR	NR	NR	NR	3/12	10/12
Pittman et al.	NR	NR	NR	NR	Oral cavity: 53/216	NR	NR	18/216	6/216	NR	3/216	NR
Breman et.al.	NR	NR	NR	NR	NR	< 100: 24/47 > 100: 23/47	1/47	NR	NR	NR	8/47	NR
Perez-Duque	NR	NR	Pelvic area and groin: 6/27	NR	Genitals: 6/27	NR	NR	NR	NR	11/27	0/27	3/27
Learned et.al.	NR	NR	NR	NR	NR	< 100: 6/8 > 100:2/8	1/11	NR	NR	NR	0/11	8/11
Mande et al.	18/21	3/21	Arms and/or hands: 18/21 Sole of the foot:15/21	NR	NR	NR	8/21	NR	NR	NR	NR	NR
Girometti et.al.	NR	48/54	Head or neck: 11/54 Arms or hands: 11/54 Pelvic area and groin: 51/54 Legs or feet: 11/54 Chest or Abdomen: 14/54	NR	Oral cavity:4/54	NR	NR	6/54	NR	NR	NR	5/54
Vaughan et al.	NR	NR	Pelvic area and groin: 1/2	NR	Oral cavity: 1/2	NR	NR	NR	NR	NR	NR	NR
Antinori et al.	NR	NR	Head or neck: 1/4 Pelvic area and groin: 3/4 Legs or feet: 3/4 Chest or Abdomen: 2/4 Sole of the foot:1/4	NR	Anal or rectal area: 2/4	NR	NR	NR	NR	NR	NR	NR
Hammerschlag et al.	NR	NR	Pelvic area and groin: 1/1 Head or neck: 1/1 Chest or Abdomen: 1/1	NR	NR	NR	NR	NR	NR	NR	NR	NR
De Nicolas-Ruanes et al.	NR	NR	Pelvic area and groin: 1/1	NR	Anal or rectal area: 1/1	NR	NR	NR	NR	1/1	NR	NR
Erez et al.	NR	NR	Entire Body: 1/1	NR	NR	NR	NR	NR	NR	1/1	NR	NR
Adler et al.	NR	7/7	Head or neck:7/7 Arms or hands: 3/7 pelvic area and groin:4/7 Legs or feet: 1/7 Chest or Abdomen:7/7 Palm:4/7 Sole of the foot:2/7	NR	NR	NR	NR	0/7	NR	2/7	0/7	7/7

*NR* not reported

Our review included nine cross-sectional studies, three cohort studies, four case reports, and three case series. Most of them were conducted in African countries (9 studies); the most extensive study was from Whitehouse et al. [31], and the smallest was from Learned et al. [37]. Case reports and case series were included only for qualitative synthesis and were the studies with the smallest populations. A total of 1958 patients from the remaining study designs were pooled in the quantitative synthesis. Males accounted for 57.6% (1129), and the age ranged from 2 days old to 69 years old. Most cross-sectional studies were at low risk of bias, and just two were at high risk. Likewise, all cohort studies, case series and case reports were at low risk of bias (Additional file 1: Table S3).

### Monkeypox clinical findings

**Fig. 2:**
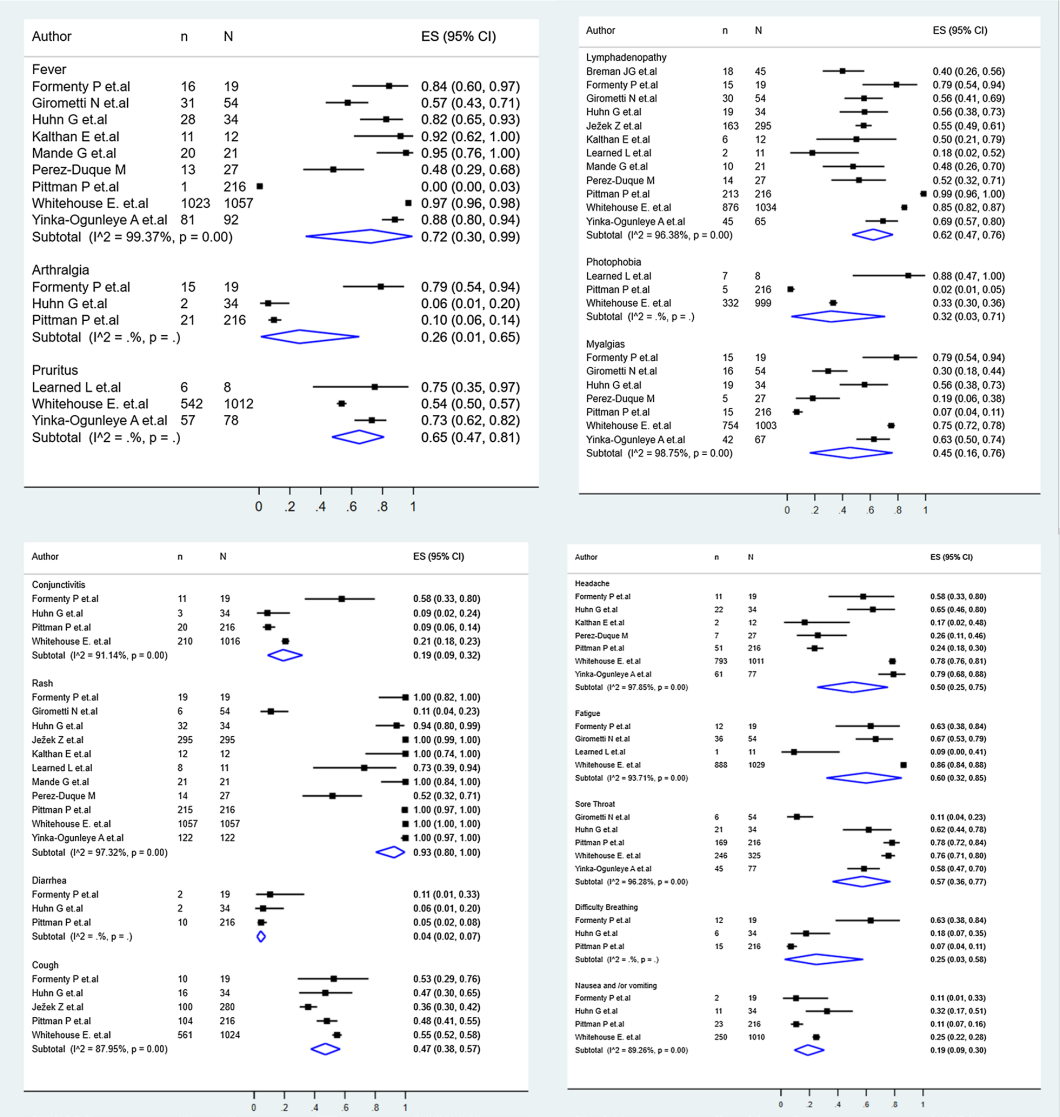
Pool prevalences forest plots of main clinical findings

Regarding the clinical manifestations, rash (93%, 95% CI: 80–100%), fever (72%, 95% CI: 30–99%), pruritus (65%, 95% CI: 47–81%), and lymphadenopathy (62%, 95% CI:47–76%), were among the most prevalent clinical manifestations (Fig. 2). Other manifestations included fatigue (60%, 95% CI: 32–85%), sore throat (57%, 95% CI: 36–77%), headache (50%, 95% CI: 25–75%), cough (47%, 95% CI: 38–57%), myalgias (45%, 95% CI: 16–76%), photophobia (32%, 95% CI: 3–71%), arthralgia (26%, 95% CI: 1–65%), difficult breathing (25%, 95% CI: 3–58%), conjunctivitis (19%, 95% CI: 9–32%), nausea/vomiting (19%, 95% CI: 9–30%), diarrhea (4%, 95% CI: 2–7%) (Fig. 2).

### Cutaneous lesions characteristics of monkeypox

**Fig. 3:**
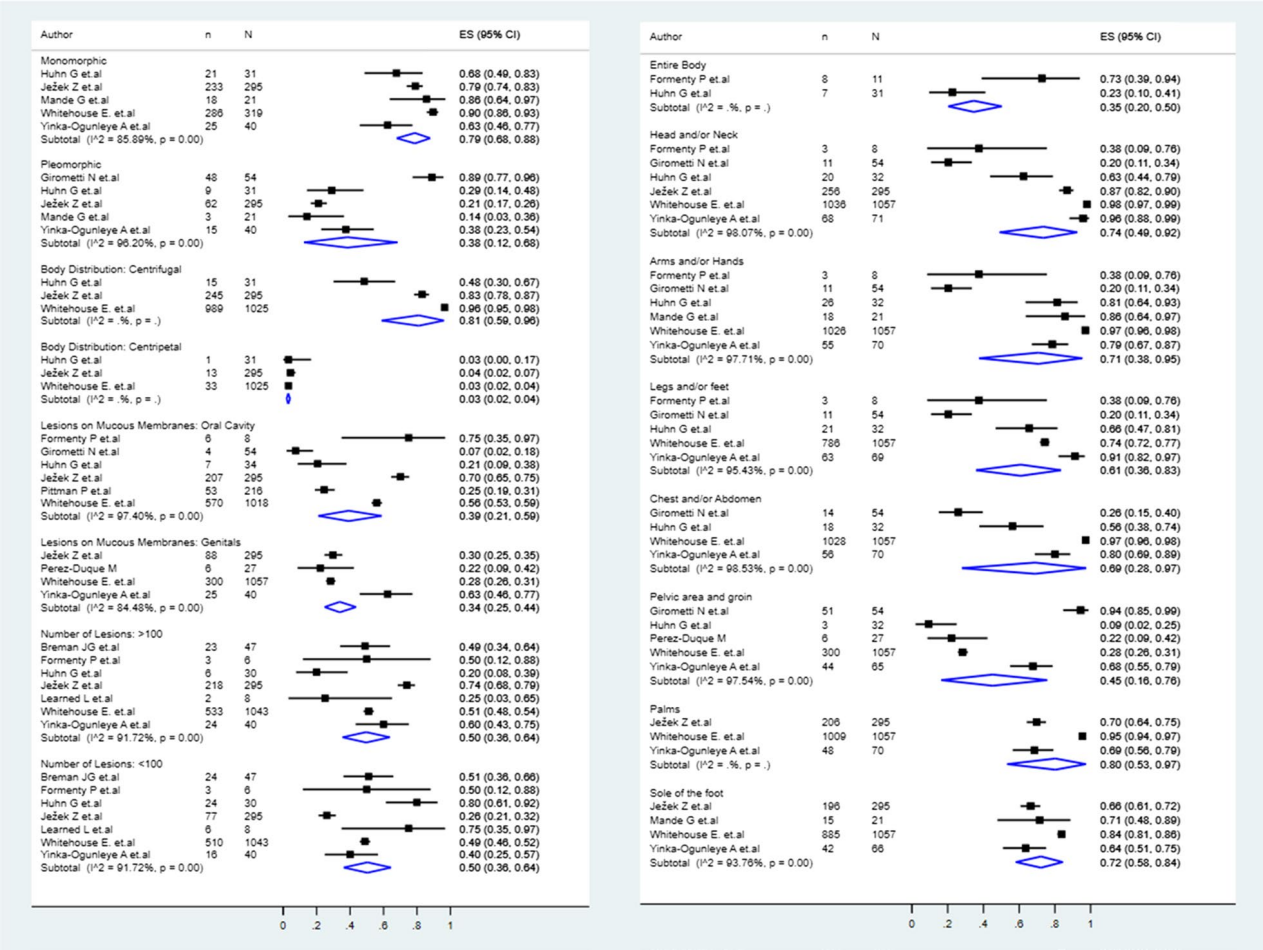
Pool prevalences forest plots of the cutaneous lesion characteristics and sites

The cutaneous lesions were mostly monomorphic (79%, 95% CI: 68–88%), instead of pleomorphic (38%, 95% CI: 12–68%), with a centrifugal distribution (81%, 95% CI: 59–96%), rather than centripetal (3%, 95% CI: 2–4%) (Fig. 3). About the number of lesions, in 50% (95% CI: 36–64%), there were < 100 lesions, and 50% (95% CI: 36–64%) had ≥ 100 lesions. Regarding the lesion distribution, these were located at head/neck (74%, 95% CI: 49–92%), hand palms (80%, 95% CI: 53–97%), foot soles (72%, 95% CI: 58–84%), arms/hands (71%, 95% CI: 38–95%), chest/abdomen (69%, 95% CI: 28–97%), legs/feet (61%, 95% CI: 36–83%), pelvic area and groins (45%, 95% CI: 16–76%), oral cavity (39%, 95% CI: 21–59%), mucosae of genitals (34%, 95% CI: 25–44%), and at the entire body (35%, 95% CI: 20–50%) (Fig. 3).

### Complications, hospitalizations and deaths associated with monkeypox

**Fig. 4:**
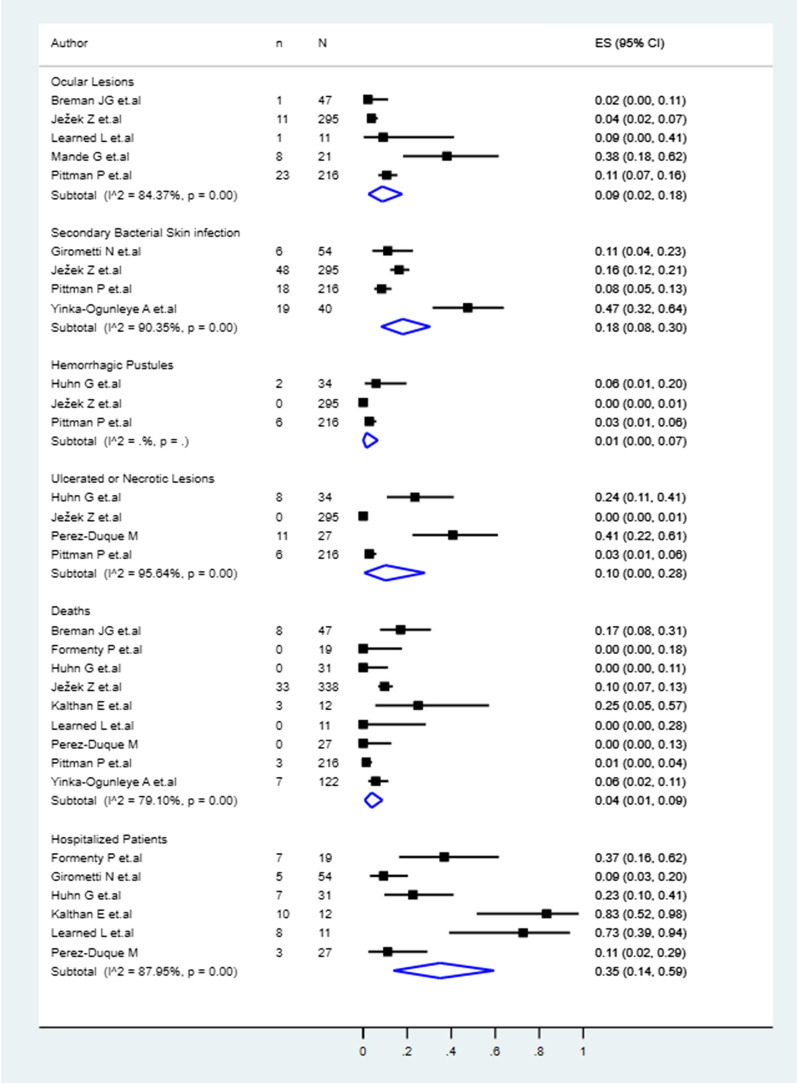
Pool prevalences forest plots of the MPX complications, hospitalisations and associated deaths

Among the patients, 9% (95% CI 2–18%) presented ocular lesions, 18% (8–30%) secondary bacterial skin infections, 1% (0–7%) haemorrhagic pustules, and 10% (0–28%) ulcerated or necrotic lesions (Fig. 4). From the patients, 35% (14–59%) were hospitalized, and 4% (1–9%) had fatal outcomes (Fig. 4).

### Subgroup analysis

In the subgroup analysis, fever, myalgias and lymphadenopathy occurred similarly between African and European patients (Additional file 1: Table S4). When comparing the frequency of rash, this was significantly higher in African studies (100%, 95% CI: 100–100%) than in European (22%, 95% CI: 14–32%) (Additional file 1: Table S1). At the same time, the distribution of the rash in the pelvic area and groins was significantly higher in European studies (75%, 95% CI: 65–84%) compared to the African (30%, 95% CI: 28–33%) (Additional file 1: Table S3). In addition, lymphadenopathy was slightly more frequent in Africa (65%, 95% CI: 47%-81%) than in Europe (54%, 95% CI: 43–65%). African patients were significantly more hospitalised (64%, 33–90%) than European (10%, 4–17%). Although no deaths have been reported in Europeans so far, all the deaths corresponded to African patients.

### Sensitivity analysis

Regarding to the sensitivity analysis (Additional file 1: Table S5), the most common clinical findings were rash (93%, 95% CI: 80–100%), cough (47%, 95% CI: 38–57%), headache (55%, 95% CI: 29–81%), fatigue (75%, 95% CI: 55–90%), throat sore (57%, 95% CI:36–77%), lymphadenopathy (67%, 95% CI: 51–81%), and pruritus (55%, 95% CI: 52–58%).Monkeypox lesions were mainly monomorphic (79%, 95% CI: 68–88%) with a centrifugal body distribution (81%, 95% CI: 59–96%), and with more than 100 lesions (52%, 95% CI: 37–66%). The most common sites of rash were head and/or neck (74%, 95% CI: 49–72%), arms and/or hands (71%, 95% CI: 38–95%), and palms (80%, 95% CI: 53–97%). The most prevalent complications were secondary bacterial skin infection (18%, 95% CI: 8–30%), hospitalization (18%, 95% CI: 8–30%), and ulcerated or necrotic lesions (10%, 95% CI: 0–28%).

## Discussion

Over the last months, more than 18,000 cases of monkeypox have been reported outside Africa, in more than 70 countries (up to July 27, 2022), for the first time in more than five decades since the virus was first detected in humans [47–50]. Monkeypox is an emerging condition outside endemic countries, rapidly spreading to multiple countries and continents due to different factors, including its potential sexual transmission [51, 52]. Preparedness at different levels, facing a new clinical disease, demands efforts in epidemiological, diagnostic, therapeutic, and preventive fields during a potential pandemic [53] threatening to spread to new territories and areas with the risk of epidemics [41, 54–59].

Clinical findings and the evolution of the disease and outcomes constitute critical knowledge that should be carefully studied when a new infectious disease emerges or reemerge. Recently, in this context of the monkeypox outbreak, several questions have been raised, including what the full spectrum of illness is, which proportion of patients present complications, need hospitalisation, and may evolve to fatal forms [60–62]. In this systematic review and meta-analysis, we tried to initially summarise clinical data on monkeypox cases published during the outbreak's first weeks. As a result, we analysed more than 1900 patients for major clinical manifestations (most of them men). Our findings are consistent with the expected.

Initial observations from imported cases [46, 63–66], confirmed by this systematic review, suggest that monkeypox's clinical presentation and evolution would differ from the findings of African studies before 2022 and the publications in non-endemic European countries. This review confirms that rash with pruritus, fever, and lymphadenopathy are critical clinical findings, but now, in connection with the potential sexual transmission or transmission due to close contact during sex, is associated more in the 2022 outbreak with rash in the pelvic area and groins (75%) compared to its frequency in African patients (30%). The rash also seems to be different between the clinical presentation in Africa and Europe, with higher frequency in endemic countries (100%), whilst relatively low in European patients (22%). In some cases, yet to be confirmed in case series and more extensive studies, patients may present with solitary or few lesions [43]. Nevertheless, it is to note that 50% of the patients show 100 or more cutaneous lesions, which corresponds to the severe skin lesion severity score of the World Health Organization [67].

Even more, and fortunately, the disease is milder in the current outbreak and outside Africa, with a rate of hospitalisation approximately of 1:6 between Europeans versus African patients, with no deaths reported so far in the ongoing outbreak outside endemic countries. This pattern is probably associated with the circulation of the milder West African clade. A recent systematic review exclusively assessing the case fatality rates (CFR%) by clades found that the CFR% for the West African clade (3.6%, 95% CI 1.7–6.8%) was significantly lower than the Central African clade (10.6%, 8.4–13.3%) [68].

Given the higher frequency of rash (93%) but also of fever (72%), the differential diagnoses of monkeypox will be broad and also dependent on the local epidemiology, including multiple vaccine-preventable diseases such measles, varicella, or even arboviral diseases in the tropics (e.g. dengue, chikungunya, Zika) [69], as well as other established sexually transmitted infections, such as syphilis and AIDS dermatitis, but also the increasing report of this zoonotic infection among people living with HIV/AIDS [18, 36, 41, 42, 70–74]. Moreover, cutaneous lesions or rash appear, as confirmed in this systematic review, head/neck, hand palms and foot soles (> 70%) [17]. Nevertheless, as discussed, genitals and even the oral cavity (in more than a third of patients) should also be explored, looking for lesions. Recently, a call was made not only for physicians but for dental surgeons to assess monkeypox as a differential and possible diagnosis during the current outbreak [75].

A critical differentiating clinical finding is lymphadenopathy (62%), which is not present in other diseases such as smallpox or varicella [76]. However, even lymphadenopathy does not differ significantly in frequency between Africans and Europeans, and probably across the years of study, confirming their importance as a clinical finding in monkeypox [77, 78].

Although most patients did not require hospitalisation (65%), multiple complications may occur, ranging from haemorrhagic pustules (1%) to secondary bacterial skin infections (18%), that may lead to fatal outcomes that have occurred among African patients, but even 10% of European patients were hospitalised.

Case–control studies and cohort studies derived from the 2022 outbreak are necessary to better define the disease's clinical evolution. Clinical characterisation by disease phases and correlation with viremia and viral DNA detection in other body fluids is currently key to understanding disease and transmission. More studies are needed to elucidate the risk factors for severe illness and death. That will allow for identifying groups most likely to have poor outcomes so that we can focus on prevention and treatment efforts. It is supposed that very young children, pregnant women [79, 80], elderly and immunocompromised persons are at higher risk of complicated disease, but this needs further assessment [81].

The laboratory abnormalities were not included in this systematic review. There is a lack of studies, more reports are needed, and only a few data are reported in recent case reports [45] and some studies before 2022 [29]. Leukopenia, thrombocytopenia, elevated blood urea nitrogen, increased hepatic transaminases (ALT/AST), and hypoalbuminemia has been reported in the past and is associated with monkeypox disease [29]. In the recent case series in the United Kingdom of imported illness from 2018 to 2021, transaminitis was reported in association with antiviral treatment in three patients using brincidofovir. Antiviral therapy is another gap in available information regarding its use and apparent efficacy and safety as a treatment for monkeypox [46, 82]. Multiple aspects of monkeypox were not addressed before the 2003 United States outbreak [83].

Our results showed that there is still a need for more comprehensive clinical studies, including short- and long-term follow-up cohort assessments. More studies from outside Africa, now that 37 non-endemic countries have reported cases are necessary to contribute to the growing volume of data, in addition to the increasing number of studies appearing in 2022 from African countries [84–86]. Even more, assessing the impact of spreading events, including the pride parties and festivals on Grand Canary island in Spain or Belgium, needs better evaluations [18, 36, 41, 42, 71–73]. More studies are also required from non-European and non-African countries, including North and Latin America, the Middle East and Oceania, regions already affected by monkeypox [8, 42, 49]. Further clinical data is crucial to elucidate the clinical spectrum of the disease. Up to now, the clinical findings are consistent regardless of report type (cross-sectional studies or case reports). However, more data are needed to define the risk factors for hospitalisation and possible fatal outcomes outside Africa. In other resource-constrained settings, different from Africa, supply chains, including those for drugs, masks and personal protection equipment, will be rechallenged, although at a lower level than COVID-19 [58].

The results of this systematic review highlight the findings that may assist clinicians anywhere in the globe in suspecting the possibility of monkeypox infection in those with recent travel to areas with the ongoing transmission or among contacts of confirmed cases, according to global and national definitions. Early recognition of cases will allow clinicians to ensure adequate clinical monitoring, supportive interventions, and preventing further transmission by implementing infection control measures [1, 2, 31, 67, 73, 87]. There is a need for prospective studies to evaluate the epidemiology, pathogenesis, duration of viral shedding, and the clinical spectrum of disease associated with this zoonotic emerging viral infection [88].

To effectively protect populations and healthcare workers in the face of the arrival and spreading of this emerging viral pathogen, constant evaluation of available evidence is essential to guide clinical suspicion, diagnosis, management and mitigation of transmission of monkeypox.

## Limitations

This review has several limitations. First, few studies are available for inclusion. Most are from Africa and Europe. It would be better to include as many studies with a broad geographic scope to understand monkeypox comprehensively. More detailed patient information, particularly regarding clinical outcomes, was unavailable in many studies at the time of analyses; however, the data in this review allow a first synthesis of the clinical characteristics of monkeypox. Finally, possible heterogeneity of the studies included should be considered for different variables. Additionally, we could not identify detailed information from the patients for many variables. In addition, the variability of the populations, including their nationality, origin or belonging to some specific ethnicity and other epidemiological aspects that they could possess, as well as the differences in the designs of the studies reviewed.

## Conclusions

Infection with the monkeypox virus is associated with significant cutaneous compromise. Therefore, most patients will not require hospitalisation. Similar to other viral pathogens, monkeypox presents a progressive course of fever in most cases. A significant distinguishing factor includes lymphadenopathy. Eliciting a history of recent travel to areas with ongoing outbreaks of this emerging pathogen or contact with a confirmed case of monkeypox should prompt clinicians to initiate isolation precautions and obtain laboratory confirmation by PCR. Additional research is needed to elucidate viral and host factors in the pathogenesis of severe and fatal infections.

## Supplementary Information

**Additional file 1: Table S1.** Search strategies. **Table S2. **Cases Definitions. **Table S3. **Quality assessment of included studies. **Table S4.** Analysis of subgroups according to continents. **Table S5.** Sensitivity analysis according to the risk of bias.
