# Supplementary material for: Clinical features, hospitalisation and deaths associated with monkeypox: a systematic review and meta-analysis
Source: Ann Clin Microbiol Antimicrob. 2022 Aug 10;21:36. doi: 10.1186/s12941-022-00527-1 (PMC9364300; doi:10.1186/s12941-022-00527-1)
Supplement: Supplementary file 1 — Additional file 1: Table S1. Search strategies. Table S2. Cases Definitions. Table S3. Quality assessment of included studies. Table S4. Analysis of subgroups according to continents. Table S5. Sensitivity analysis according to the risk of bias. [file 12941_2022_527_MOESM1_ESM.docx]

**Additional file 1**

**Table S1.** Search strategies.

| **Source** | **PubMed** |
| --- | --- |
| **Search** | **Formula** |
|  | Monkeypox virus [MH] OR Monkeypox [MH] OR “monkeypox*” [TIAB] OR (“monkey*” [TIAB] AND “pox*” [TIAB]) OR “chimpanzeepox*” [TIAB] |
| **Source** | **Scopus** |
| **Search** | **Formula** |
|  | TITLE-ABS-KEY (“monkeypox*” OR (“monkey*” W/3 “pox*”) OR “chimpanzeepox*”) |
| **Source** | **Web of Science** |
| **Search** | **Formula** |
|  | TI=(“monkeypox*” OR (“monkey*” NEAR/3 “pox*”) OR “chimpanzeepox*”) OR AB=(“monkeypox*” OR (“monkey*” NEAR/3 “pox*”) OR “chimpanzeepox*”) OR AK=(“monkeypox*” OR (“monkey*” NEAR/3 “pox*”) OR “chimpanzeepox*”) OR KP=(“monkeypox*” OR (“monkey*” NEAR/3 “pox*”) OR “chimpanzeepox*”) OR TS=(“monkeypox*” OR (“monkey*” NEAR/3 “pox*”) OR “chimpanzeepox*”) |
| **Source** | **Embase** |
| **Search** | **Formula** |
|  | 'monkeypox'/exp OR (“monkeypox*” OR (“monkey*” NEAR/3 “pox*”) OR “chimpanzeepox*”):ti OR (“monkeypox*” OR (“monkey*” NEAR/3 “pox*”) OR “chimpanzeepox*”):ab OR (“monkeypox*” OR (“monkey*” NEAR/3 “pox*”) OR “chimpanzeepox*”):kw |
| **Source** | **OVID** |
| **Search** | **Formula** |
|  | (monkeypox* OR (monkey* adj3 pox*) OR chimpanzeepox*).ti. OR (monkeypox* OR (monkey* adj3 pox*) OR chimpanzeepox*).ab. OR (monkeypox* OR (monkey* adj3 pox*) OR chimpanzeepox*).kw. |

**Table S2.** Cases Definitions.

| **Author** | **Probable Case** | **Confirmed Case** |
| --- | --- | --- |
| Formenty P et.al | Any person from the outbreak zone evaluated by a clinician who sought treatment during September 2005–January 2006 for fever (>37.5°C) had a vesicular-pustular rash similar to that shown in a WHO reference photograph and had an epidemiologic link to a confirmed case | Cases were confirmed based on any of the following laboratory findings from clinically derived specimens: -ELISA (show immunoglobulin M against orthopoxvirus)  - viral identification by real-time PCR  -Isolation of MPXV |
| Huhn G et.al | Any person who had illness onset <21 days of exposure to MPXV who experienced fever (>37.4°C) and vesicular pustular rash, or rash (potentially uncharacterised) plus had immunoglobulin (IgM) to orthopoxvirus. | Cases were confirmed based on any of the following laboratory findings from clinically derived specimens: MPXV isolation, detection of MPXV-specific nucleic acid signatures, positive electron microscopy findings, or positive immunohistochemical findings (the last 2 in the absence of other orthopoxvirus virus exposures). |
| Yinka-Ogunleye A et.al | Any suspected case in whom laboratory testing could not be done but who could be epidemiologically linked with a confirmed case | Cases were confirmed based on any of the following laboratory findings from clinically derived specimens: viral identification by real-time PCR, antibody detection, or viral isolation |
| Whitehouse E. et.al | NR | Cases were confirmed based on any of the following laboratory findings from clinically derived specimens: viral identification by real-time PCR or isolation of MPXV in culture from ≥1 specimen |
| Ježek Z et.al | NR | NR |
| Kalthan E et.al | NR | NR |
| Pittman P et.al |  | Viral identification by real-time PCR |
| Breman JG et.al |  | Cases were confirmed based on any of the following laboratory findings from clinically derived specimens:  -MPXV isolation, detection of MPXV-specific nucleic acid signatures  -Positive electron microscopy findings or positive immunohistochemical findings (the last 2 in the absence of other orthopoxvirus virus exposures). |
| Perez-Duque M | A person of any age who meets the suspected case criteria AND one or more of the following conditions:  -contact with a suspected, probable or confirmed case of MPXV infection within 21 days before the onset of symptoms  -a person who had multiple or anonymous sexual partners within 21 days before symptom onset  -hospital admission because of a clinical condition consistent with a suspected case  -travel history to MPX-endemic countries within 21 days before symptom onset. | In a clinical sample, a person with a laboratory-confirmed MPX infection (real-time PCR-positive test result and/or nucleotide sequencing). |
| Learned L et.al | Meets one of the epidemiologic criteria : Exposure to an African endemic animal species, individuals of which have been identified with elevated levels of orthopoxvirus-reactive antibodies or exposure to a probable or confirmed human case of monkeypox AND  Fever and vesicular-pustular rash with onset of first sign or symptoms <21 days after last exposure meeting epidemiologic criteria or if rash is present but the type is not described, demonstrates elevated levels of IgM antibodies reactive with orthopox virus between at least days 7–56 after rash onset | Cases were confirmed on the basis of any of the following laboratory findings from clinically derived specimens:  -Isolation of monkeypox virus in culture or  -Demonstration of monkeypox virus DNA by polymerase chain reaction testing of a clinical specimen or  -Demonstration of presence of orthopoxvirus in tissue using immunohistochemical testing methods, or electron microscopy, in the absence of exposure to another orthopoxvirus. |
| Mande G et.al | NR | viral identification by real-time PCR |
| Girometti N et.al | NR | viral identification by real-time PCR |
| Vaughan et.al | NR | viral identification by real-time PCR |
| Antinori A et.al | NR | viral identification by real-time PCR |
| Hammerschlag Y et.al | NR | viral identification by real-time PCR |
| De Nicolas-Ruanes B et.al | NR | viral identification by real-time PCR |
| Reynolds M et.al | NR | viral identification by real-time PCR |
| Erez N et.al | NR | A person with a laboratory-confirmed MPX infection (immunofluorescence assay, tissue culture, or ELISA) |
| Adler H et.al | NR | viral identification by real-time PCR |

NR: NOT REPORTED

**Table S3.** Quality assessment of included studies.

|  | **NEWCASTLE - OTTAWA QUALITY ASSESSMENT SCALE FOR CROSS-SECTIONAL STUDIES** | | | | | | | | | | |
| --- | --- | --- | --- | --- | --- | --- | --- | --- | --- | --- | --- |
| **STUDY** | | **SELECTION** | | | | **COMPARABILITY** | **OUTCOME** | | |  |  |
|  | | **Representativeness of the sample** | **Sample size** | **Non-respondents** | **Ascertainment of the exposure (risk factor)** | **The subjects in different outcome groups are comparable, based on the study design or analysis. Confounding factors are controlled.**  **Maximum : ☆☆** | **Assessment of outcome**  **Maximum : ☆☆** | **Statistical test** | | **SCORE** | **Evidence quality** |
| **Formenty P et.al** | | ☆ | ☆ | ☆ | ☆ | ☆☆ | ☆ | ☆ | | *8* | Low risk of bias |
| **Huhn G et.al** | | ☆ | ☆ | ☆ | ☆ | ☆ | ☆ | ☆ | | *7* | Low risk of bias |
| **Yinka-Ogunleye A et.al** | | ☆ | ☆ | ☆ | ☆ | ☆☆ | ☆ | ☆ | | *8* | Low risk of bias |
| **Ježek Z et.al** | | ☆ | ☆ | ☆ | ☆ | ☆ | ☆ | ☆ | | *7* | Low risk of bias |
| **Kalthan E et.al** | | ☆ | ☆ |  | ☆ | ☆ | ☆ | ☆ | | *6* | High risk of bias |
| **Breman JG et.al** | | ☆ | ☆ | ☆ | ☆ | ☆☆ | ☆ | ☆ | | *8* | Low risk of bias |
| **Perez-Duque M et.al** | | ☆ | ☆ | ☆ | ☆ | ☆ | ☆ | ☆ | | *7* | Low risk of bias |
| **Learned L et.al** | | ☆ | ☆ |  | ☆ | ☆ | ☆ | ☆ | | *6* | High risk of bias |
| **Mande G et.al** | | ☆ | ☆ | ☆ | ☆ | ☆ | ☆ | ☆ | | *7* | Low risk of bias |
|  | **NEWCASTLE - OTTAWA QUALITY ASSESSMENT SCALE FOR COHORT STUDIES** | | | | | | | | | | |
| **STUDY** | | **SELECTION** | | | | **COMPARABILITY** | **OUTCOME** | |  |  |  |
|  | | **Representativeness of the exposed cohort** | **Selection of the non-exposed cohort** | **Ascertainment of exposure** | **Demonstration that outcome of interest was not present at start of study** | **Comparability of Cohorts on the Basis of the Design or Analysis Maximum : ☆☆** | **Assessment of outcome** | **Was follow-up long enough for outcomes to occur** | **Adequacy of follow up of cohorts** | **SCORE** | **Evidence quality** |
| **Whitehouse E. et.al** | | ☆ | ☆ | ☆ | ☆ | ☆ | ☆ | ☆ | ☆ | 8 | Low risk of bias |
| **Pittman P et.al** | | ☆ | ☆ | ☆ | ☆ | ☆ | ☆ | ☆ | ☆ | 8 | Low risk of bias |
| **Girometti N et.al** | | ☆ | ☆ | ☆ | ☆ | ☆ | ☆ | ☆ | ☆ | 8 | Low risk of bias |

|  | | **Joanna Briggs Institute Critical Appraisal Checklist for Case Reports** | | | | | | | | | | | | | | | | | | | | |
| --- | --- | --- | --- | --- | --- | --- | --- | --- | --- | --- | --- | --- | --- | --- | --- | --- | --- | --- | --- | --- | --- | --- |
| **STUDY** | | **Were patient’s demographic characteristics clearly described?** | | **Was the patient’s history clearly described and presented as a timeline?** | **Was the current clinical condition of the patient on presentation clearly described?** | | **Were diagnostic tests or assessment methods and the results clearly described?** | | **Was the intervention(s) or treatment procedure(s) clearly described?** | | **Was the post-intervention clinical condition clearly described?** | | **Were adverse events (harms) or unanticipated events identified and described?** | | **Does the case report provide takeaway lessons?** | | **SCORE** | | **Evidence quality** | | | |
| **Hammerschlag Y et.al** | | ☆ | | ☆ | ☆ | |  | | ☆ | | ☆ | | ☆ | | ☆ | | 7 | | Low risk of bias | | | |
| **De Nicolas-Ruanes B et.al** | | ☆ | | ☆ | ☆ | | ☆ | | ☆ | | ☆ | | ☆ | | ☆ | | 8 | | Low risk of bias | | | |
| **Reynolds M et.al** | | ☆ | | ☆ | ☆ | | ☆ | | ☆ | | ☆ | | ☆ | | ☆ | | 8 | | Low risk of bias | | | |
| **Erez N et.al** | | ☆ | | ☆ | ☆ | | ☆ | | ☆ | | ☆ | | ☆ | | ☆ | | 8 | | Low risk of bias | | | |
|  | | **Joanna Briggs Institute Critical Appraisal Checklist for Case series** | | | | | | | | | | | | | | | | | | | | |
| **STUDY** | ***Were there clear criteria for inclusion in the case series?*** | | ***Was the condition measured in a standard, reliable way for all participants included in the case series?*** | | | ***Were valid methods used for identification of the condition for all participants included in the case series?*** | | ***Did the case series have consecutive inclusion of participants?*** | | ***Did the case series have complete inclusion of participants?*** | | ***Was there clear reporting of the demographics of the participants in the study?*** | | ***Was there clear reporting of clinical information of the participants?*** | | ***Were the outcomes or follow up results of cases clearly reported?*** | | ***Was there clear reporting of the presenting site(s)/clinic(s) demographic information?*** | | ***Was statistical analysis appropriate?*** | ***SCORE*** | **Evidence quality** |
| **Vaughan et.al** | ☆ | | ☆ | | | ☆ | | ☆ | |  | | ☆ | | ☆ | | ☆ | | ☆ | | ☆ | 9 | Low risk of bias |
| **Antinori A et.al** | ☆ | | ☆ | | | ☆ | | ☆ | |  | | ☆ | | ☆ | | ☆ | | ☆ | | ☆ | 9 | Low risk of bias |
| **Adler H et.al** | ☆ | | ☆ | | | ☆ | | ☆ | | ☆ | | ☆ | | ☆ | | ☆ | | ☆ | | ☆ | 10 | Low risk of bias |

**Table S4.** Analysis of subgroups according to continents

| 1. **AFRICA** | | |
| --- | --- | --- |
| ***Clinical manifestations*** | ***ES(95% CI)*** | ***I^2^ ; p value*** |
| Conjunctivitis | 0.23(0.1-0.39) | Not possible to calculate |
| Rash | 1(1-1) | I^2^ =61.67% , p=0.01 |
| Diarrhea | 0.04(0.02-0.07) | Not possible to calculate |
| Cough | 0.47(0.36-0.58) | I^2^ =90.92% , p=0.00 |
| Headache | 0.52(0.22-0.82) | I^2^ =98.45% , p=0.00 |
| Fatigue | 0.56(0.13-0.94) | Not possible to calculate |
| Sore Throat | 0.72(0.63-0.81) | Not possible to calculate |
| Difficulty Breathing | 0.25(0.03-0.58) | Not possible to calculate |
| Nausea and/or vomiting | 0.16(0.06-0.29) | Not possible to calculate |
| Lymphadenopathy | 0.65(0.47-0.81) | I^2^ =97.03% , p=0.00 |
| Photophobia | 0.32(0.03-0.71) | Not possible to calculate |
| Myalgia | 0.54(0.12-0.93) | I^2^ =99.31% , p=0.00 |
| Fever | 0.77(0.2-1) | I^2^ =99.59% , p=0.00 |
| Arthralgia | 0.13(0.03-0.18) | Not possible to calculate |
| Pruritus | 0.65(0.47-0.81) | Not possible to calculate |
| ***Characteristics of the lesions*** | ***ES(95% CI)*** | ***I^2^ ; p value*** |
| Monomorphic | 0.81(0.69-0.9) | I^2^ =87.42% , p=0.00 |
| Pleomorphic | 0.24(0.14-0.36) | Not possible to calculate |
| Body distribution : Centrifugal | 0.94(0.93-0.96) | Not possible to calculate |
| Body distribution : Centripetal | 0.03(0.03-0.05) | Not possible to calculate |
| Lesions on mucous membranes : Oral Cavity | 0.54(0.34-0.74) | I^2^ =97.43% , p=0.00 |
| Lesions on mucous membranes : Genitals | 0.36(0.26-0.47) | Not possible to calculate |
| Number of lesions : >100 | 0.55(0.41-0.69) | I^2^ =91.08% , p=0.00 |
| Number of lesions : <100 | 0.45(0.31-0.59) | I^2^ =91.08% , p=0.00 |
| ***Rash site*** | ***ES(95% CI)*** | ***I^2^ ; p value*** |
| Head and/or Neck | 0.9(0.77-0.99) | I^2^ =95.47% , p=0.00 |
| Arms and/or Hands | 0.82(0.58-0.98) | I^2^ =93.53% , p=0.00 |
| Legs and/or feet | 0.76(0.56-0.92) | Not possible to calculate |
| Chest and/or Abdomen | 0.97(0.96-0.98) | Not possible to calculate |
| Pelvic area and groin | 0.3(0.28-0.33) | Not possible to calculate |
| Palms | 0.8(0.53-0.97) | Not possible to calculate |
| Sole of the foot | 0.72(0.58-0.84) | I^2^ =93.76% , p=0.00 |
| ***Complications*** | ***ES(95% CI)*** | ***I^2^ ; p value*** |
| Ocular lesions | 0.09(0.02-0.18) | I^2^ =84.37% , p=0.00 |
| Secondary Bacterial Skin infection | 0.21(0.08-0.37) | Not possible to calculate |
| Hemorrhagic pustules | 0.01(0-0.02) | Not possible to calculate |
| Ulcerated or necrotic Lesions | 0.01(0-0.02) | Not possible to calculate |
| Deaths | 0.06(0.01-0.12) | I^2^ =81.41% , p=0.00 |
| Hospitalized patients | 0.64(0.33-0.9) | Not possible to calculate |
| 1. **EUROPE** | | |
| ***Clinical manifestations*** | ***ES(95% CI)*** | ***I^2^ ; p value*** |
| Lymphadenopathy | 0.54(0.43-0.65) | Not possible to calculate |
| Fever | 0.54(0.43-0.65) | Not possible to calculate |
| Myalgia | 0.26(0.17-0.36) | Not possible to calculate |
| Rash | 0.22(0.14-0.32) | Not possible to calculate |
| ***Rash site*** | ***ES(95% CI)*** | ***I^2^ ; p value*** |
| Pelvic area and groin | 0.75(0.65-0.84) | Not possible to calculate |
| ***Complications*** | ***ES(95% CI)*** | ***I^2^ ; p value*** |
| Hospitalized patients | 0.1(0.04-0.17) | Not possible to calculate |

**Table S5.** Sensitivity analysis according to the risk of bias

| 1. **LOW RISK OF BIAS** | | |
| --- | --- | --- |
| ***Clinical manifestations*** | ***ES(95% CI)*** | ***I^2^ ; p value*** |
| Conjunctivitis | 0.19(0.09-0.32) | I^2^ =91.14% , p=0.00 |
| Rash | 0.93(0.8-1) | I^2^ =97.79% , p=0.00 |
| Diarrhea | 0.04(0.02-0.07) | Not possible to calculate |
| Cough | 0.47(0.38-0.57) | I^2^ =87.95% , p=0.00 |
| Headache | 0.55(0.29-0.81) | I^2^ =98.12% , p=0.00 |
| Fatigue | 0.75(0.55-0.9) | Not possible to calculate |
| Sore Throat | 0.57(0.36-0.77) | I^2^ =96.28% , p=0.00 |
| Difficulty Breathing | 0.25(0.03-0.58) | Not possible to calculate |
| Nausea and/or vomiting | 0.19(0.09-0.3) | I^2^ =89.26% , p=0.00 |
| Lymphadenopathy | 0.67(0.51-0.81) | I^2^ =96.8% , p=0.00 |
| Photophobia | 0.26(0.23-0.28) | Not possible to calculate |
| Myalgia | 0.45(0.16-0.76) | I^2^ =98.75% , p=0.00 |
| Fever | 0.7(0.25-0.99) | I^2^ =99.45% , p=0.00 |
| Arthralgia | 0.26(0.01-0.65) | Not possible to calculate |
| Pruritus | 0.55(0.52-0.58) | Not possible to calculate |
| ***Characteristics of the lesions*** | ***ES(95% CI)*** | ***I^2^ ; p value*** |
| Monomorphic | 0.79(0.68-0.88) | I^2^ =85.89% , p=0.00 |
| Pleomorphic | 0.38(0.12-0.68) | I^2^ =96.2% , p=0.00 |
| Body distribution : Centrifugal | 0.81(0.59-0.96) | Not possible to calculate |
| Body distribution : Centripetal | 0.03(0.02-0.04) | Not possible to calculate |
| Lesions on mucous membranes : Oral Cavity | 0.39(0.21-0.59) | I^2^ =97.4% , p=0.00 |
| Lesions on mucous membranes : Genitals | 0.34(0.25-0.44) | I^2^ =84.48% , p=0.00 |
| Number of lesions : >100 | 0.52(0.37-0.66) | I^2^ =92.82% , p=0.00 |
| Number of lesions : <100 | 0.48(0.34-0.63) | I^2^ =92.82% , p=0.00 |
| ***Rash site*** | ***ES(95% CI)*** | ***I^2^ ; p value*** |
| Entire Body | 0.35(0.2-0.5) | Not possible to calculate |
| Head and/or Neck | 0.74(0.49-0.72) | I^2^ =98.07% , p=0.00 |
| Arms and/or Hands | 0.71(0.38-0.95) | I^2^ =97.71% , p=0.00 |
| Legs and/or feet | 0.61(0.36-0.83) | I^2^ =95.43% , p=0.00 |
| Chest and/or Abdomen | 0.69(0.28-0.97) | I^2^ =98.53% , p=0.00 |
| Pelvic area and groin | 0.45(0.16-0.76) | I^2^ =97.54% , p=0.00 |
| Palms | 0.8(0.53-0.97) | Not possible to calculate |
| Sole of the foot | 0.72(0.58-0.84) | I^2^ =93.76% , p=0.00 |
| ***Complications*** | ***ES(95% CI)*** | ***I^2^ ; p value*** |
| Ocular lesions | 0.09(0.02-0.19) | I^2^ =88.1% , p=0.00 |
| Secondary Bacterial Skin infection | 0.18(0.08-0.3) | I^2^ =90.35% , p=0.00 |
| Hemorrhagic pustules | 0.01(0.00-0.07) | Not possible to calculate |
| Ulcerated or necrotic Lesions | 0.1(0.00-0.28) | I^2^ =95.64% , p=0.00 |
| Deaths | 0.04(0.01-0.08) | I^2^ =81.99% , p=0.00 |
| Hospitalized patients | 0.18(0.08-0.3) | I^2^ =61.87% , p=0.05 |
